# Design and conduct of Xtreme Everest 2: An observational cohort study of Sherpa and lowlander responses to graduated hypobaric hypoxia

**DOI:** 10.12688/f1000research.6297.1

**Published:** 2015-04-10

**Authors:** Edward Gilbert-Kawai, Adam Sheperdigian, Thomas Adams, Kay Mitchell, Martin Feelisch, Andrew Murray, Mark Peters, Grace Gilbert-Kawai, Hugh Montgomery, Denny Levett, Rajendra Kumar, Michael Mythen, Michael Grocott, Daniel Martin

**Affiliations:** 1 University College London Centre for Altitude Space and Extreme Environment Medicine, UCLH NIHR Biomedical Research Centre, Institute of Sport and Exercise Health, London, W1T 7HA, UK; 2 Integrative Physiology and Critical Illness Group, Faculty of Medicine, University Hospital Southampton NHS Foundation Trust, Southampton, SO16 6YD, UK; 3 Anaesthesia and Critical Care Research Unit, University Hospital Southampton NHS Foundation Trust, Southampton, SO16 6YD, UK; 4 NIHR Southampton Respiratory Biomedical Research Unit, Southampton, CB2 3EG, UK; 5 Department of Physiology, Development & Neuroscience, University of Cambridge, Cambridge, CB2 3EG, UK; 6 Critical Care Group Portex Unit, UCL, Institute of Child Health, London, WC1N 1EH, UK; 7 Nepal Health Research Council, Kathmandu, Nepal

**Keywords:** Hypoxia, Critical care, Sherpa, High altitude, Microcirculation, Nitric Oxide, Mitochondria

## Abstract

**Objective: **Oxygen availability falls with ascent to altitude and also as a consequence of critical illness. Because cellular sequelae and adaptive processes may be shared in both circumstances, high altitude exposure (‘physiological hypoxia’) assists in the exploration of the response to pathological hypoxia. We therefore studied the response of healthy participants to progressive hypobaric hypoxia at altitude. The primary objective of the study was to identify differences between high altitude inhabitants (Sherpas) and lowland comparators.

**Methods:** We performed an observational cohort study of human responses to progressive hypobaric hypoxia (during ascent) and subsequent normoxia (following descent) comparing Sherpas with lowlanders. Studies were conducted in London (35m), Kathmandu (1300m), Namche Bazaar (3500m) and Everest Base Camp (5300m). Of 180 healthy volunteers departing from Kathmandu, 64 were Sherpas and 116 were lowlanders. Physiological, biochemical, genetic and epigenetic data were collected. Core studies focused on nitric oxide metabolism, microcirculatory blood flow and exercise performance. Additional studies performed in nested subgroups examined mitochondrial and metabolic function, and ventilatory and cardiac variables. Of the 180 healthy participants who left Kathmandu, 178 (99%) completed the planned trek. Overall, more than 90% of planned testing was completed. Forty-four study protocols were successfully completed at altitudes up to and including 5300m. A subgroup of identical twins (all lowlanders) was also studied in detail.

**Conclusion:** This programme of study (Xtreme Everest 2) will provide a rich dataset relating to human adaptation to hypoxia, and the responses seen on re-exposure to normoxia. It is the largest comprehensive high altitude study of Sherpas yet performed. Translational data generated from this study will be of relevance to diseases in which oxygenation is a major factor.

## Introduction

A wide range of pathologies can lead to deterioration of a patient such that they require admission to a critical care unit. Critically ill patients are therefore a heterogeneous group of severely ill individuals, in whom hypoxaemia (a lack of oxygen in arterial blood) is common and may subsequently lead to cellular hypoxia ^1^. The resulting cellular dysfunction may cause organ damage, and in some cases death ^2^. Traditional management of established critical illness, based on efforts to increase convective oxygen delivery through augmented cardiac output, haemoglobin concentration and oxygenation, does not appear to improve outcomes ^3^. Additionally excessive oxygen therapy may cause harm ^4, 5^. Consequently the role of alternative potential therapeutic targets such as the microcirculation, mitochondrial activity and nitric oxide (NO) are becoming increasingly more popular as alternative strategies ^6– 9^. Permissive hypoxaemia has also been proposed as a viable option for critically ill patients, to avoid the harms associated with invasive methods of restoring normoxaemia in the critically ill ^10^.

Understanding the mechanisms of human hypoxic adaptation in the context of critical illness is difficult. The wide range of underlying diseases and the complexity of treatment interactions with physiology make structured experiments challenging. The study of human responses to hypoxia occurring as a consequence of hypobaria at high altitude may be used as an alternative method of exploring elements of the pathophysiology of critical illness ^1, 11, 12^. Studying healthy individuals progressively exposed to hypobaric hypoxia defines beneficial adaptive responses and may identify candidate pathways in the critically ill. Animal models, often proposed as being a suitable alternative to large-scale field studies, fail to match the complexity of human physiology in the critically ill patient ^13^, and discordance between multiple models has been described ^14^.

In 2007, the University College London (UCL) Centre for Altitude, Space and Extreme Environment Medicine (CASE Medicine) conducted the largest study of human volunteers at high altitude, Caudwell Xtreme Everest (CXE) ^12, 15^. Resulting data have emphasized the need for studying the microcirculation ^16– 18^, NO formation and metabolism ^19^, and mitochondrial biology ^20– 22^. A further research programme (Xtreme Everest 2 (XE2)) was therefore proposed to address this ^23^. XE2 was designed to study the physiological (especially microcirculatory, mitochondrial, NO-related metabolic and epigenetic) responses to hypobaric hypoxia in native lowlanders, and compare them to those in Sherpas ^23^. Sherpas are descended from an ancestral high altitude population resident on the Tibetan plateau for over 500 generations ^24^. Such high altitude populations show evidence of genetic selection ^25– 29^ that may underpin their anecdotally reported extraordinary tolerance to hypoxia. Their phenotype may therefore hold the key to successful hypoxic adaptation in humans.

We describe the design and conduct of XE2, our approach to high altitude translational research, and discuss the strengths and weaknesses of this programme of investigation.

## Protocol

XE2 (December 2012 to May 2013) was undertaken by the Xtreme Everest Oxygen Research Consortium (XE-ORC), which comprises a partnership between the UK’s UCL CASE Medicine, the University of Southampton Centre for Human Integrative Physiology (CHiP), and Duke University Medical Centre (DUMC) in the USA.

## Ethical approval

The study design, risk management plan and protocols were approved (in accordance with the declaration of Helsinki) both by the UCL Research Ethics Committee and the Nepal Health Research Council (NHRC).

## Enrolment and informed consent

All potential participants, recruited through word of mouth and advertisement, were given written information about the study. Our Nepali Research Leader (RKBC) translated this locally in Nepal. Opportunities for questions, in person or over the telephone, were offered at multiple stages in both countries, and all participants submitted written consent for participation in the studies. At all stages throughout the expedition, independent translators were present to allow for communication between Sherpas and investigators.

## Medical screening

Eligible participants were lowland children aged 8 to 17 years, or adults (aged 18 years or above) of either lowland or Sherpa origin. For the lowland participants, an independent expert experienced in mountain medicine identified those fit to travel to altitude by reviewing a health-questionnaire (supplementary material) and telephone interviews as required. The forms of those selected were then screened by the expedition Chief Medical Officer (GGK) to confirm fitness both to travel to altitude, and to participate in research. Potential participants considered ‘at risk’ were either telephoned or reviewed in person by GGK. Where appropriate, and with permission, the participant’s medical practitioner was contacted. Those with significant cardiac or respiratory disease (e.g. severe chronic obstructive airway disease, ischemic heart disease with angina, or symptomatic heart failure) were excluded. For the Sherpa subjects, two doctors performed a pre-recruitment screening interview in Nepali based on the health-questionnaire. One doctor was a local Nepali (RKBC) and one an expert experienced in mountain medicine and XE2 investigator (DL/MG) who confirmed fitness for travel to altitude and research. As all participants would be undergoing cardiopulmonary exercise testing (CPET), additional exclusion criteria based on the American Thoracic Society/American College of Chest Physicians guidelines for clinical exercise testing were also adhered to ^30^.

## Medical safety

At each laboratory, an independent Medical Officer (MO) was responsible for decisions relating to the participants involvement with research protocols, the administration of medication and ascent or descent from their current altitude. Participants were recommended not to self-medicate, but to consult either the site MO, or their trek leader when between laboratory sites. In order to standardize medical care, clear guidelines for common altitude and non-altitude related conditions were formulated prior to departure, and adhered to. Medication use was recorded by the individual (in a standardized daily diary) and by the trek leader or MO.

## Study participants

In total, 187 participants were selected for inclusion in the study and underwent baseline testing. Sherpas were defined as direct descendants (for two generations) of Nepali Sherpas, drawn from communities in the Solukhumbu and Rowaling valleys. Sherpa participants were recruited by word of mouth through local community contacts and were required to have evidence of two generations of all Sherpa ancestors (parents and grand-parents). A detailed altitude history was then taken from all Sherpa participants including their altitude in utero, at birth, during childhood, in adulthood, and for the 12 months preceding XE2. Lowlanders were recruited in the UK; all were born and lived below 1000m, and were not from a native high altitude population (e.g. Tibetan, Andean, Ethiopian). They included European, American and South African participants. Lowlanders were divided into four cohorts:

- ***Core*** - eligible for all studies; eight of these participants were monozygotic twins (four pairs), for a pilot epigenetics study.
- ***Nitrate metabolism*** - allocated only to be involved in a study of whole body NO production.
- ***Investigators*** - who conducted the studies and remained at altitude for the duration of the expedition permitting studies of exposure to chronic hypoxia.
- ***Children*** - who took part in the Young Everest 2 Study (YES2) expedition linked to XE2 in which children ascended to Namche Bazaar (NB) to participate in a programme of non-invasive studies. YES2 will not be discussed further in this manuscript.

Potential investigators were sourced through word of mouth at CASE Medicine events and interviewed prior to their appointment (EGK, DM, KM).

All participants were required to provide baseline information that included details of previous altitude exposure and the occurrence of any altitude-related illnesses. Importantly, within the *Core* and *Investigator* cohorts, some selected participants had previously taken part in CXE (2007), thereby allowing the evaluation of consistency in the individual response to an identical high altitude exposure.

Of the 187 participants that were selected for the study and underwent baseline testing, 180 departed for high altitude investigations ( Figure 2). Of the seven participants that withdrew from the study prior to ascent to altitude, six lowlanders withdrew in London (LDN) (three for medical reasons), and one Sherpa withdrew in Kathmandu (KTM). Additionally, in LDN one person did not meet the American Thoracic Society/American College of Chest Physicians guidelines for clinical exercise testing, and was withdrawn from CPET prior to departure. Baseline characteristics for each cohort are listed in Table 1.

Of the 104 lowlanders (children excluded), and 64 Sherpas leaving KTM, 102 (98%) and 64 (100%) reached Everest base camp (EBC) respectively. Of the two who did not reach EBC, one dropped out at NB, and the other at Pheriche due to gastrointestinal and cardiovascular problems respectively. All the *Investigators* reached their allocated laboratories, however, one left EBC early for medical reasons, and two for personal reasons. Two investigators left NB early for personal reasons, and one investigator left NB early to move to EBC. One investigator also arrived late at EBC.

## Study setting

Baseline ‘normoxic’ (35m) data for lowlander *Core* and *Investigator* groups were collected at The London Clinic Hospital, from 3 ^rd^ December 2012 to 25 ^th^ January 2013. Sherpa baseline testing was performed in KTM (1300m; 4 ^th^ March to 16 ^th^ April 2013). Between baseline testing and trek ascent, participants remained below 3000m in order to avoid hypoxic exposure prior to the expedition.

## Ascent profile

**Figure 1.**
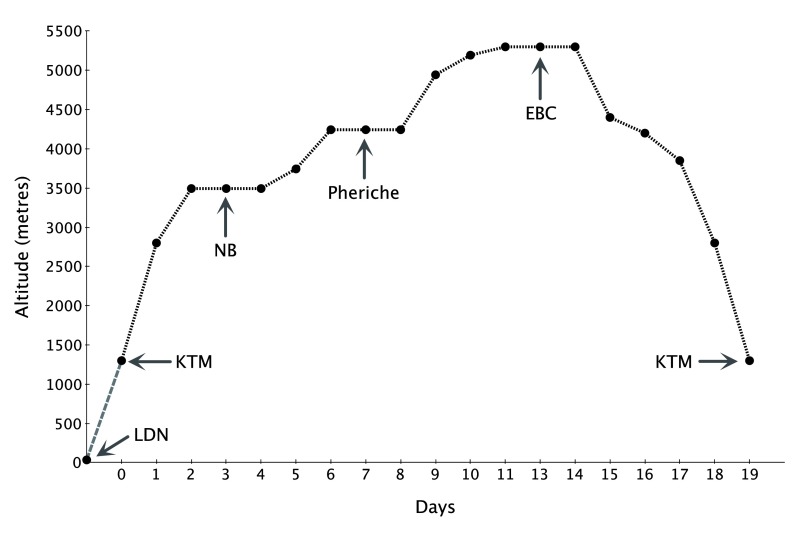
Ascent and descent profile for XE2 Everest Base Camp trek. Key: LDN = London, KTM = Kathmandu, NB = Namche Bazaar, EBC = Everest Base Camp.

Participants trekked in groups of up to 14. All lowlanders flew to KTM and spent one night there prior to flying to Lukla (2800m). Similarly, Sherpas flew from KTM to Lukla at the beginning of the trek. In order to ensure that study participants were exposed to an identical pattern of hypoxia exposure, all participants followed an identical ascent and descent profile ( Figure 1). High altitude field laboratories were situated at NB (3500m) and EBC (5300m). The KTM laboratory was also used for descent testing for participants following their return from EBC. Barometric pressure, temperature, and humidity data were recorded twice daily at each laboratory and are summarized in Table 2.

**Figure 2.**
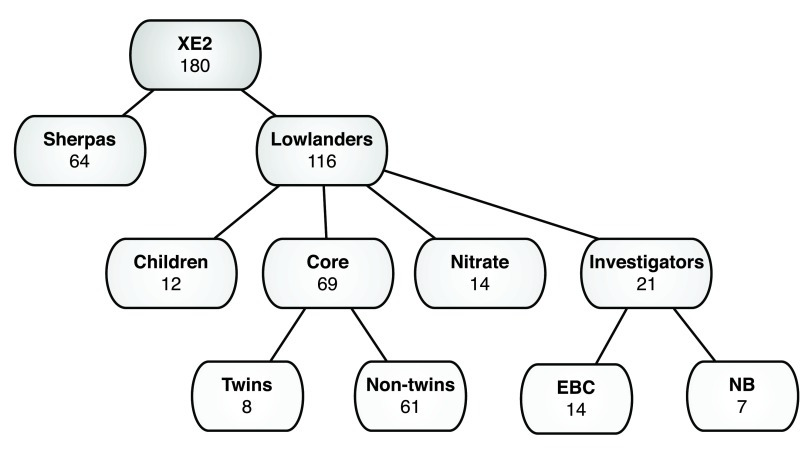
Break down of study cohorts showing number of participants tested. Key: NB = Namche Bazaar, EBC = Everest Base Camp.

**Table 1.** The mean (±SD) baseline characteristics for each cohort (excluding the children cohort). Key: NB = Namche Bazaar, EBC = Everest Base Camp.

COHORT	AGE	HEIGHT (cm)	WEIGHT (kg)	GENDER (% male)
**Core** **(n = 69)**	41.3 (13.9)	171 (10)	71.1 (13.5)	39%
**EBC Investigators** **(n = 14)**	30.2 (5.3)	177 (10)	75.7 (17.3)	60%
**NB Investigators** **(n = 7)**	30.0 (8.5)	177 (6)	69.2 (7.2)	71%
**Nitrate Study** **(n = 14)**	43.0 (17)	172 (9)	81.7 (16.4)	36%
**Sherpas** **(n = 64)**	27.9 (6.9)	160 (6)	61.3 (8.9)	47%

**Table 2.** Mean (±SD) altitude, barometric pressure, temperature and humidity at each XE2 laboratory. Key: LDN = London, KTM = Kathmandu, NB = Namche Bazaar, EBC = Everest Base Camp.

Laboratory	Altitude (m)	Barometric pressure (Millibar)	Temperature (°C)	Humidity (%)
**LDN**	35	1006 (2.1)	16.9 (1.8)	35.4 (6.5)
**KTM**	1300	868 (3.7)	23.8 (3.4)	47.4 (15.7)
**NB**	3500	665 (2.4)	13.9 (3.1)	72.1 (8.1)
**EBC**	5300	530 (2.4)	12.9 (8.2)	37.8 (17.5)

The ascent to EBC from KTM was completed over 11 days with incorporated rest days built in to the schedule to reduce the likelihood of acute mountain sickness (AMS). This ascent profile was identical to the CXE profile both because it was proven safe with minimal participant dropouts and because it would allow comparison of data between the two expeditions ^12^.

The *Investigator* cohort underwent a similar ascent profile to the rest of the participants, but then remained *in situ* at their relevant laboratories for six weeks prior to descent. By using repeated testing at serial intervals, we were able to study the effects of both acute and chronic hypoxia on these participants. It should also be noted that upon their arrival at EBC, the *Investigators* were required to construct the laboratory and set up equipment, and consequently testing was started two days after arrival.

## Laboratory testing and studies

Upon arrival at each laboratory participants all were given a camp safety and science brief, and individualized timetables. Within these, in an attempt to minimize diurnal variations in physiological responses, participants were tested for each study at the same time at each site. Furthermore as many studies required abstinence from caffeine and food for specific periods, fasting periods and meal times were clearly highlighted for each individual. Within each laboratory, the timetable was adhered to and subjects were tested for particular studies on either day one or day two. These specific days were kept constant throughout the trek so as to control for the effects of acute acclimatization and responses over time. Because of the potential interaction between some studies, not every participant underwent all of the studies. The XE2 research portfolio, detailing core studies, additional studies and those in common with CXE, are highlighted in Table 3 and Table 4.

**Table 3.** Additional studies conducted on XE2, highlighting at which lab and in which cohorts.

Study	Baseline (LDN/KTM)	NB	EBC	Descent
**Haemoglobin and oxygen carriage**				
Haemoglobin mass analysis	✔ ^Ψ Φ Ω^	✖	✔ ^Ψ Φ Ω^	✔ ^Ψ Φ Ω^
**Exercise**				
Exercise efficiency and economy ^^^	✔ ^Ψ Φ Ω^	✔ ^Ψ Φ Ω^	✔ ^Ψ Φ Ω^	✔ ^Ψ Φ Ω^
Oxygen uptake kinetics	✔ ^Ψ Φ Ω^	✔ ^Ψ Φ Ω^	✔ ^Ψ Φ Ω^	✔ ^Ψ Φ Ω^
Constant power tests / critical power	✔ ^Ω^	✖	✔ ^Ω^	✔ ^Ω^
**Nitric Oxide**				
Whole body NO production	✔ ^ξ^	✖	✔ ^ξ^	✖
**Mitochondria and metabolic**				
Mitochondrial function in skeletal muscle	✔ ^Ψ Φ Ω^	✖	✔ ^Ψ Φ Ω^	✔ ^Ψ Φ Ω^
Oral glucose tolerance test	✔ ^Ψ Φ Ω^	✖	✔ ^Ψ Φ Ω^	✔ ^Ψ Φ Ω^
Metabolomic sampling	✔ ^Ψ Φ Ω^	✔	✔ ^Ψ Φ Ω^	✔ ^Ψ Φ Ω^
Skeletal muscle mass ^^^	✔ ^Ω^	✖	✔ ^Ω^	✔ ^Ω^
Catecholamine sampling	✔ ^Ψ Φ Ω^	✔ ^Ψ Φ Ω^	✔ ^Ψ Φ Ω^	✔ ^Ψ Φ Ω^
Endogenous steroid sampling	✔ ^Ψ Φ Ω^	✔ ^Ψ Φ Ω^	✔ ^Ψ Φ Ω^	✔ ^Ψ Φ Ω^
**Ventilation**				
Hypoxic ventilatory response	✔ ^Ψ Φ Ω^	✖	✔ ^Ψ Φ Ω^	✔ ^Ψ Φ Ω^
Dejours procedure	✖	✖	✔ ^Ψ Φ Ω^	✖
**Upper airway**				
Extra (supra)-oesophageal reflux	✔ ^Ψ Φ Ω^	✖	✔ ^Ψ Φ Ω^	✖
Laryngoscopy ^^^	✔ ^Ψ Φ Ω^	✖	✔ ^Ψ Φ Ω^	✖
Nasal saccharine testing and naso-mucociliary clearance ^^^	✔ ^Ψ Φ Ω^	✖	✔ ^Ψ Φ Ω^	✖
Nasal secretion sampling	✔ ^Ψ Φ Ω^	✖	✔ ^Ψ Φ Ω^	✖
Acoustic rhinometry	✔ ^Ψ Φ Ω^	✖	✔ ^Ψ Φ Ω^	✖
Sino-nasal outcome testing questionnaire	✔ ^Ψ Φ Ω^	✖	✔ ^Ψ Φ Ω^	✔ ^Ψ Φ Ω^
**Pulmonary**				
Extra-vascular lung water analysis	✔ ^Ψ Φ Ω^	✖	✔ ^Ψ Φ Ω^	✔ ^Ψ Φ Ω^
**Cardiovascular**				
Blood pressure variation	✔ ^Ψ Φ Ω^	✔ ^Ψ Φ Ω^	✔ ^Ψ Φ Ω^	✖
Heart rate variability	✔ ^Ψ Φ Ω^	✔ ^Ψ Φ Ω^	✔ ^Ψ Φ Ω^	✖
**Neocytolysis**	✔ ^Ψ Φ Ω^	✖	✔ ^Ψ Φ Ω^	✖
**Iron studies**	✔ ^Ψ Φ Ω^	✔ ^Ψ Φ Ω^	✔ ^Ψ Φ Ω^	✔ ^Ψ Φ Ω^
**Arterial blood gas analysis ^^^**	✔ ^Ω^	✖	✔ ^Ω^	✖
**Proteomic sampling**	✔ ^Ψ Φ Ω^	✖	✔ ^Ψ Φ Ω^	✖
**Telomere analysis**	✔ ^Ψ Φ Ω^	✖	✔ ^Ψ Φ Ω^	✖

^^^ = Also studied on CXE Conducted in: ^Ψ^ = Sherpas, ^Φ^ = Lowlanders (Core), ^Ω^ = Investigators, ^ξ^ = Nitrate

**Table 4.** Core studies conducted on XE2, highlighting at which lab and in which cohorts.

Study	Baseline (LDN/KTM)	NB	EBC	KTM descent
**Symptom assessment**				
Daily diary ^^^	✔ ^Ψ Φ Ω^	✔ ^Ψ Φ Ω^	✔ ^Ψ Φ Ω^	✔ ^Ψ Φ Ω^
Acute mountain sickness questionnaire	✔ ^Φ Ω^	✖	✔ ^Φ Ω^	✔ ^Φ Ω^
**Haemoglobin and oxygen carriage**				
Estimated arterial oxygen content ^^^	✔ ^Ψ Φ Ω^	✔ ^Ψ Φ Ω^	✔ ^Ψ Φ Ω^	✔ ^Ψ Φ Ω^
**Exercise**				
Maximum exercise capacity ^^^	✔ ^Ψ Φ Ω^	✔ ^Ψ Φ Ω^	✔ ^Ψ Φ Ω^	✔ ^Ψ Φ Ω^
**Nitric Oxide**				
Saliva sampling	✔ ^Ψ Φ Ω^	✔ ^Ψ Φ Ω^	✔ ^Ψ Φ Ω^	✔ ^Ψ Φ Ω^
Exhaled breath condensate sampling	✔ ^Ψ Φ Ω^	✔ ^Ψ Φ Ω^	✔ ^Ψ Φ Ω^	✔ ^Ψ Φ Ω^
Exhaled nitric oxide analysis	✔ ^Ψ Φ Ω^	✔ ^Ψ Φ Ω^	✔ ^Ψ Φ Ω^	✔ ^Ψ Φ Ω^
Oral nitrate reduction test	✔ ^Ψ Φ Ω^	✔ ^Ψ Φ Ω^	✔ ^Ψ Φ Ω^	✔ ^Ψ Φ Ω^
Plasma sampling ^^^	✔ ^Ψ Φ Ω^	✔ ^Ψ Φ Ω^	✔ ^Ψ Φ Ω^	✔ ^Ψ Φ Ω^
Urine sampling	✔ ^Ψ Φ Ω^	✔ ^Ψ Φ Ω^	✔ ^Ψ Φ Ω^	✔ ^Ψ Φ Ω^
**Microcirculation**				
Venous plethysmography	✔ ^Ψ Φ Ω^	✔ ^Ψ Φ Ω^	✔ ^Ψ Φ Ω^	✔ ^Ψ Φ Ω^
Incident dark field imaging ^^^	✔ ^Ψ Φ Ω^	✖	✔ ^Ψ Φ Ω^	✔ ^Ψ Φ Ω^
Laser Doppler flowmetry	✔ ^Ψ Φ Ω^	✔ ^Ψ Φ Ω^	✔ ^Ψ Φ Ω^	✔ ^Ψ Φ Ω^
Near-infrared spectroscopy	✔ ^Ψ Φ Ω^	✖	✔ ^Ψ Φ Ω^	✔ ^Ψ Φ Ω^
**Genetic and epigenetic**				
Genetic sampling ^^^	✔ ^Ψ Φ Ω^	✖	✖	✖
Epigenetic sampling	✔ ^Ψ Φ Ω^	✔ ^Ψ Φ Ω^	✔ ^Ψ Φ Ω^	✔ ^Ψ Φ Ω^
**Ventilation**				
Spirometry ^^^	✔ ^Ψ Φ Ω^	✔ ^Ψ Φ Ω^	✔ ^Ψ Φ Ω^	✔ ^Ψ Φ Ω^

^^^ = Also studied on CXE Conducted in: ^Ψ^ = Sherpas, ^Φ^ = Lowlanders (Core), ^Ω^ = Investigators

## Biological sample storage and transport

Blood, urine, saliva and exhaled breath condensate samples were all kept in -40°C freezers at each site. Muscle biopsy specimens (LDN, KTM and EBC) were stored in liquid nitrogen (-196°C), and then shipped to the UK on dry ice. All samples were brought from the field to KTM by helicopter and then repatriated to the UK on dry ice (-78°C).

## Outline of analysis plan

Data analysis will follow a predetermined strategy of:

i) Description of phenotypes for each of the studies as outlined in Table 3 and Table 4 including plasma biomarkers and metabolomic analyses. This will include comparison with data obtained during a matched ascent in the CXE 2007 study (e.g. intra-individual comparison of the phenotypes from individuals who were participants in XE2 and CXE) as well as sub-group analyses (e.g. twins).

ii) Comparison of phenotypic adaptations between Sherpas and lowlanders during ascent and descent. We hypothesise that Sherpas will have a phenotype at baseline that is better adapted to hypoxia in comparison to lowlanders, that lowlanders ascending to altitude will tend to converge on the Sherpa phenotype, and that the Sherpa baseline phenotype will be less perturbed by altitude exposure than the lowlander baseline phenotype.

iii) Integrative analysis of genotype-epigenome-transcriptome-phenome across multiple datasets (XE2, CXE). The XE2 dataset will contribute to the accumulated Extreme Everest BioResource acquired during a number of high altitude research expeditions. Data will be incorporated into our bespoke comprehensive database, which enables linkage of all data elements with meta-data describing the provenance of each data item through the use of semantic web technology. Key questions will be raised in an iterative manner, driven both by a priori hypotheses and subsequently by data mining focused on the results obtained by unbiased outputs from individual ‘omics analyses.

Results from this study will be disseminated in peer-reviewed journals, on the Xtreme Everest website ( www.xtreme-everest.co.uk) at scientific conferences and at public meetings.

## Discussion and conclusions

We have characterized many features of the human response to progressive hypobaric hypoxia in a cohort of 180 individuals; 44 individual studies being safely conducted over four locations up to 5300m. In addition, the response to relative re-oxygenation was studied. The standardized ascent protocol ensured that differences between participants reflected inter-individual variability in hypoxic adaptation, as opposed to variability in hypoxic exposure. In matching the 2007 CXE ascent profile, the data from the two studies may also be combined to create a single cohort. The parallel study of lowlanders and highlanders will permit the identification of beneficial phenotypic adaptations and genetic alteration (and their relationships) in these groups. The study of investigators exposed to sustained (six weeks) hypoxia facilitated study of immediate and longer-term adaptive processes.

The slow ascent profile minimized symptoms of AMS, increasing the number of participants successfully reaching EBC and available for the study. Despite good medical care, gastrointestinal illness may have occasionally confounded results. However, the application of standardized medical protocols, with detailed documentation of illness and medication, will ensure this can be accounted for. Whilst the expedition was promoted through word of mouth and public advertisement, the very nature of the trek itself meant that participants were of a self-selected nature as enthused to undertake a rigorous trek. They may thus not be representative of the general population. Laboratories in Nepal were temporary as opposed to permanent structures. Temperature and pressure fluctuations, both having the potential to confound data obtained, were recorded on a daily basis ( Table 2). Whilst we attempted to maintain a constant temperature between all laboratories through the use of heaters, we accept that any measured differences may confound the results.

We believe that the wealth of data obtained from XE2 will aid our understanding of the human adaptive response to hypoxia, offering insights that may be of value to patients suffering from sub-acute hypoxemia as a result of critical illness.
