# Supplementary material for: Design and conduct of Xtreme Everest 2: An observational cohort study of Sherpa and lowlander responses to graduated hypobaric hypoxia
Source: F1000Res. 2015 Apr 10;4:90. [Version 1] doi: 10.12688/f1000research.6297.1 (PMC4448741; doi:10.12688/f1000research.6297.1)
Supplement: Peer review history

# Peer review: Referee response for version 1

**DOI:** 10.5256/f1000research.6754.r8809

**Published:** 2015-05-28

**Authors:** Jean-Paul Richalet

**Affiliations:** 1 Laboratoire “Hypoxie & Poumon” EA2363, Université Paris 13, Sorbonne Paris Cité, Bobigny, France

The article by Gilbert-Kawai and co-workers presents the design of Xtreme Everest 2 expedition, which looks like a continuation of the first Caudwell Xtreme Everest expedition led in 2007.

They present a complete description of protocols and groups of subjects. Part of the experiments concern classical domains already explored by many scientific expeditions (exercise capacity, hemglobin, ventilation, heart rate variability, etc..). Some aspects are more interesting and original, such as epigenetics, microcirculation, study of twins and Sherpas, although some important information are lacking concerning, for example the tissue studied for epigenetics, if Sherpas are involved in the epigenetic study, etc.

Altogether, we can hope that a lot of new data will be available for a better understanding of physiological responses to hypoxia, and a greater number of publications than for the first Xtreme Everest expedition is expected.

The argument that was often put forward to justify this extremely complex and costly type of experimental design is that it might be of great value for patients suffering from critical illnesses associated with hypoxia. I think this argument is unnecessary. The study of normal persons exposed to hypoxia is, by itself, very interesting and worthwhile, and does not need any justification. Applications to patients of what will be found is probable but impossible to predict.

I have read this submission. I believe that I have an appropriate level of expertise to confirm that it is of an acceptable scientific standard.

# Peer review: Referee response for version 1

**DOI:** 10.5256/f1000research.6754.r8288

**Published:** 2015-05-05

**Authors:** James E. Clark

**Affiliations:** 1 Centre of Human & Aerospace Physiological Sciences and British Heart Foundation Excellence Centre, Cardiovascular Division, St Thomas’ Hospital, King’s College London, London, UK

This article described the planning and undertaking of the study design for the Xtreme Everest 2 research expedition which looks at Sherpa and lowlander responses to graduated hypobaric hypoxia.

The authors are experienced in this kind on study having already worked on Xtreme Everest 1 study some years ago. The objectives are stated clearly and the rationale for the study described with the underlying physiology. Part of the difficulty of this kind of study is the recruitment process. It would be hard to recruit widely for this kind of expedition as the subjects are usually self-selecting, those people who are keen to explore and ascent to altitude. However the authors have explained their recruitment methods and the study participant information has been shown.

The study itself is fairly straightforward and includes a gradual, measured ascent to altitude and a series of tests undertaken prior, during and after ascent and the descent following the expedition. Within the basic study there is a cohort of subjects taking part in the "nitrate" study, to investigate whether dietary nitrate supplementation has an impact on hypoxic tolerance and adaptation to altitude. 

A comprehensive table of outcomes and measurements is given which shows the magnitude of the study and the way in which the investigators aimed to separate the workload between cohorts. The measures taken are widespread and range from skeletal muscle mitochondrial function to blood composition and numerous cardiovascular and respiratory functional analysis for which this group of investigators have considerable experience. 

The data from this study will be beneficial to the field of hypoxia, altitude performance and will give us a better understanding into the adaptations found in those living at altitude. This will translate into patient care in the clinical scenario as hypoxia and oxygen delivery can affect many in intensive care.

I have read this submission. I believe that I have an appropriate level of expertise to confirm that it is of an acceptable scientific standard.
